# Semantic Representation of Robot Manipulation with Knowledge Graph

**DOI:** 10.3390/e25040657

**Published:** 2023-04-14

**Authors:** Runqing Miao, Qingxuan Jia, Fuchun Sun, Gang Chen, Haiming Huang, Shengyi Miao

**Affiliations:** 1 School of Automation, Beijing University of Posts and Telecommunications, Beijing 100876, China; 2 Institute for Artificial Intelligence, Tsinghua University, Beijing 100084, China; 3 College of Electronics and Information Engineering, Shenzhen University, Shenzhen 518060, China

**Keywords:** robot manipulation, knowledge graph, representation learning, graph neural network

## Abstract

Autonomous indoor service robots are affected by multiple factors when they are directly involved in manipulation tasks in daily life, such as scenes, objects, and actions. It is of self-evident importance to properly parse these factors and interpret intentions according to human cognition and semantics. In this study, the design of a semantic representation framework based on a knowledge graph is presented, including (1) a multi-layer knowledge-representation model, (2) a multi-module knowledge-representation system, and (3) a method to extract manipulation knowledge from multiple sources of information. Moreover, with the aim of generating semantic representations of entities and relations in the knowledge base, a knowledge-graph-embedding method based on graph convolutional neural networks is proposed in order to provide high-precision predictions of factors in manipulation tasks. Through the prediction of action sequences via this embedding method, robots in real-world environments can be effectively guided by the knowledge framework to complete task planning and object-oriented transfer.

## 1. Introduction

It is crucial to apply a strong understanding of relevant skills and task-planning capabilities to service robots, whether in daily life or in industrial-assembly scenarios. Classic task and motion planning (TAMP) [1] relies heavily, often entirely, on predefined planning domains, symbolic rules, and complex strategy searches. This leads to high costs and an inability to process tasks in a way that reflects human cognition and semantics. In general, all the limitations and constraints must be known before starting a task; otherwise, failures in efficient transfer and in the ability to adapt to changing task scenarios may occur

In relation to this problem, the past decade has witnessed rapid developments in the field of robot manipulation, especially in knowledge-based methods of representation and task planning. These enlightening paradigms enable robots to acquire manipulative task-related knowledge from human knowledge. However, knowledge is a higher-dimensional form of organization than data; discrete and structured characteristics also suffer from difficulties in directly describing continuous manipulation data. Therefore, most of the existing knowledge-based robot-manipulation-representation methods focus on static-object information and usually fail to elucidate reasonable decoupling between different factors. Concretely, the descriptions of tasks, actions, and skills are flat and chaotic. Furthermore, only rule-based symbolic calculations are considered during the processes of querying and reasoning.

Efficient and reasonable representations of complex manipulation knowledge require the consideration of both continuous and discrete data, as well as static and dynamic factors. Real-time responses and continuous interactive updates are also necessary for knowledge systems to handle new tasks. Additionally, the reasonable modeling of manipulation processes and the extraction of semantic information are prerequisites for precise inferences and planning.

In this paper, a semantic representation framework for robot manipulation is introduced, based on a knowledge graph, to represent human and robotic knowledge about various manipulation tasks. The knowledge-representation model covers six factors of manipulation via a multi-layer structure: scene, object, agent, task, action, and skill. The knowledge-representation system consists of a high-level knowledge base and a low-level graph database, divided into three mutually independent and interactive modules: ontology, template, and instance. In order to add sufficient task knowledge to the knowledge base, we extracted knowledge from text datasets and external knowledge bases centered on manipulation tasks. In total, we obtained 10,768 triples, including 936 entities and 21 relations.

A deep-learning method was designed for robot-manipulation-task planning and object-oriented transfer, in which a graph neural network was utilized as an encoder and a knowledge-graph-embedding model was set as a decoder. Embedded representations of entities and relations in the knowledge base were generated, robot-manipulation factors were converted into feature vectors, and the action sequences of the robot in new object-manipulation tasks were predicted. The superiority of our knowledge-graph-embedding method was validated through comparative experiments, with rates of potential-relation-prediction accuracy of 65.7% for Hits@1, 77.4% for Hits@3, and 87.9% for Hits@10. Finally, the role of knowledge-graph-based semantic representation in robot-manipulation-task planning and object-oriented transfer was evaluated by real-world experiments. With the predicted action sequences, the UR5 robot achieved a 91.7% action-sequence-prediction accuracy and an 81.8% execution accuracy in one hundred and twenty trials, including twenty-four different real-world tasks in three categories and with eighteen objects. The main contributions of this paper can be outlined as follows:

- A knowledge framework is introduced, which includes a multi-layer robot-manipulation knowledge-representation model, including six hierarchically decoupled layers: scene, object, agent, task, action, and skill. Additionally, a multi-module knowledge-representation system, consisting of ontology, template, and instance modules, is presented.
- Pursuant to the knowledge framework, a task-centric semantic-knowledge-extraction method is proposed, which extracts multi-domain knowledge from a life-guide dataset and an external knowledge base.
- Taking the knowledge framework as the cornerstone, a knowledge-graph-embedding method based on graph neural networks is presented, and its superiority is validated by comparative experiments.
- The effects of our knowledge framework and embedding method are evaluated through a real-world robot. Robot-manipulation-task planning and object-oriented transfer are achieved.

The remaining parts of this paper are organized as follows. Section 2 introduces related works on knowledge-based robot manipulation and knowledge-graph embedding. Section 3 provides a brief description of the overall framework. Section 4 elaborates on the robotic-manipulation knowledge-representation model and system. Section 5 introduces the semantic knowledge extraction for task-centric manipulation. Section 6 presents the knowledge-graph-embedding method based on graph neural networks. The experimental results and analysis are given in Section 7. Finally, the conclusion is presented in Section 8.

## 2. Related Works

### 2.1. Knowledge-Based Robot Manipulation

The application of service robots in manipulation tasks is usually divided into three levels: the planning level, which includes task analysis and task planning; the strategy level, which includes the motion planning of an action primitive; and the control level, which includes the execution process of the robot’s hardware. Specifically, the knowledge-based method mainly focuses on the planning level, and fruitful results have been achieved with knowledge-graph-based robot-knowledge-representation systems. RoboEarth [2,3] was the first attempt to explore a knowledge-sharing system among robots, which mainly involved storing point clouds, CAD models, and object images in robot manipulation, which can enable robots to build semantic maps, which are required for daily tasks. On this basis, a robot can fully utilize cyberspace in its living space [4]. KnowRob [5] acquires an obtaining object and environmental information from the web and stores it in a knowledge-processing system that can be queried using Prolog, providing the knowledge required for service robots to perform daily manipulation tasks. KnowRob2 [6] improves the ability to acquire and reason knowledge based on KnowRob. RoboBrain [7] focuses on data collection. The knowledge engine stores different modalities of data, including symbols, natural language, tactile senses, robot trajectories, and visual features. Subsequently, they are connected to produce rich, heterogeneous graph representations. Perception and Manipulation Knowledge (PMK) [8] involves the formalization and implementation of a standardized ontology framework to extend robots’ abilities related to manipulation tasks requiring task and motion planning (TAMP). Centering around scalability and responsiveness, TRPO [9] designs corresponding task-planning algorithms, starting from three categories of ontology knowledge: task, environment, and robot. Furthermore, AKB-48 [10] constructs a large-scale knowledge graph of articulated objects, which includes 2037 3D articulated object models from 48 categories in the real world. RoboKG [11] constructs a knowledge base specifically for performing grasping-based manipulation tasks, assisting the robot in the prediction of factors related to grasping, such as which component of the object to grasp, which end effector to use, and how much force to apply during grasping.

These knowledge-representation systems each have at their core a large-scale static-knowledge graph, which takes static objects in robot manipulation as the main description objects, and the description of manipulation behavior remains at the level of the simple recording of high-level tasks or low-level motion parameters. In addition, the architectures of these knowledge-representation systems are either excessively singular and flat, or overly chaotic and disorderly, resulting in high query complexity. In contrast, our knowledge-representation model achieves hierarchical decoupling between differentmanipulation factors, especially the characterization of dynamic tasks, actions, and skill relations. We implement a layered semantic representation of manipulation knowledge based on the latest graph database, Neo4j [12]. From upstream knowledge extraction to downstream knowledge queries and task planning, we construct a prototype knowledge framework for robot manipulation.

### 2.2. Knowledge-Graph Embedding

The knowledge graph originated in semantic networks [13] and is a typical form of structured knowledge representation. It represents structures of facts, consisting of entities, relations, and semantic descriptions [14]. Entities can be real-world objects and abstract concepts; relations represent the relations between entities. The semantic descriptions of entities and their relations contain categories and properties with clear meanings. Mature knowledge-graph schemes include the language-knowledge base WordNet [15], the world-knowledge base Freebase [16], Wikidata [17], DBpedia [18], and ConceptNet [19,20].

The goal of knowledge-graph embedding is to represent the semantic knowledge of the research object as a dense, low-dimensional, real-valued vector using machine learning. The low-dimensional vector representation obtained by embedding is a distributed representation [21]. The embedding of knowledge graphs is focused on entities and relations in the knowledge base, in contrast to mapping, which considers spatial, temporal, and logical dimensions in the Internet of Things [22]. By mapping entities or relations into a low-dimensional vector space, the semantic information can be represented, and the complex semantic associations between entities and relations can be efficiently calculated. This is critical for both knowledge updates and reasoning.

The current mainstream knowledge-graph-embedding method is the translation model. TransE [23] regards relations as translation operations from the head entity to the tail entity in low-dimensional vector space. TransH [24] solves the problem of TransE’s inability to handle 1–n, n–1, and n–n relations well. TransR [25] replaces the projection vector in TransH with a projection matrix.

Graph neural networks mainly solve the problem of non-Euclidean space, which is naturally suitable for the graph structures of knowledge graphs. Nevertheless, classical graph convolutional networks [26] are isomorphic graphs, in which all the edges in the network share the same weight and cannot adapt to different types of relation in knowledge graphs. Therefore, a heterogeneous graph-embedding method [27] is proposed to calculate the weights of different relations separately to deal with multi-relation graph data. In our work, the knowledge base that describes robot manipulation tasks has a hierarchical structure that differs from those of other knowledge bases. Therefore, the knowledge-graph-embedding methods described above feature major limitations in robot manipulation-task planning and object-oriented transfer.

## 3. Overall Framework

**Figure 1:**
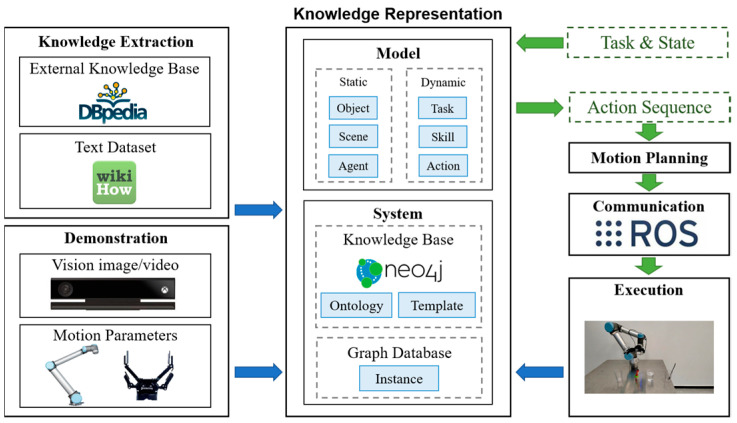
The overall knowledge-based framework of robot manipulation, including a representation model and system and knowledge-extraction and task-planning processes.

The framework of this work is shown in Figure 1. The blue arrows indicate the flow of knowledge. Our knowledge-representation model consists of a static-scene layer, an object layer, an agent layer, a dynamic-task layer, an action layer, and a skill layer. Ontology and template knowledge are obtained from external knowledge bases and text datasets and stored in the knowledge base of the knowledge-representation system. Multi-modal data on manipulation demonstrations, which may be in real or simulated environments, are stored as instances in the graph database of the knowledge-representation system. The green arrows represent the process of robot manipulation. For a given task, we query the task templates in the knowledge base and return prior knowledge, which mainly consists of semantic action sequences. After motion planning with the RRT-based planner [28] and communication with the ROS system, the robot executes the task. Upon the completion of the task, the new instance generated from the current task is added to the graph database to enable data feedback and closed-loop task execution. This is the basic operational mode of the knowledge-representation system. With knowledge-graph embedding, it is possible for robots to perform manipulation tasks and object-oriented transfer. Overall, the knowledge framework realizes the semantic and hierarchical representation of robot manipulation, which is of great significance for robot manipulation-task planning and transfer.

## 4. Manipulation-Knowledge Representation

Before building the knowledge-representation model and system, two questions need to be clarified: (1) Which factors need to be represented in manipulation? (2) How can different types of knowledge be described and stored? To answer these questions, in Section 4.1, we introduce a multi-layer knowledge-representation model to represent various factors in manipulation and their relations. In Section 4.2, we describe a multi-module knowledge-representation system to adapt to the decoupling of different types of knowledge.

### 4.1. Multi-Layer Manipulative-Knowledge-Representation Model

This section first formally defines the manipulation model from the perspective of semantic cognition.

(1) M=(L,H,A,O,T,S)

There are six factors in manipulation: in the scene L, the agent H needs to perform a set of action primitives $A=\left\{a_{1},a_{2},\ldots ,a_{n}\right\}$ facing a group of objects $O=\left\{o_{1},o_{2},\ldots ,o_{n}\right\}$ to complete the task T, as well as applying and learning skills S in different tasks. The scene L, the agent R, and the object O are static factors, which means that their knowledge is independent and stable and can be described as common-sense knowledge. The task T, the action A, and the skill S are dynamic factors, which means that their knowledge is complex and changeable, and that their content changes according to changes in other factors.

#### 4.1.1. Scene

The scene layer is used to describe the external spatial information when manipulation occurs and is divided into two categories: coarse-grained environment and fine-grained area. The environment description mainly focuses on indoor spaces in which human activities take place, such as kitchens, rooms, and workshops. The areas are targeted at manipulation robots and describe the relevant parameters of the manipulation platform, such as lighting and tabletop materials, as well as precise area divisions.

#### 4.1.2. Object

The object layer is used to describe the physical objects involved in manipulation. To provide a more precise knowledge-based definition of objects, the common-sense knowledge base and the object-knowledge base AKB-48 [10] are comprehensively considered, and the properties of objects in different manipulation scenarios are annotated. An object’s semantic information is first recorded in the knowledge-base ontology, including its name, concept description, and hypernym. Next, the physical properties are recorded. These include mass, size, material, form, color, and packing. Finally, information regarding visual appearance, including 3D models, multi-view RGB-D snapshots, and basic RGB images, are recorded.

#### 4.1.3. Agent

The agent layer is used to describe the execution subject in manipulation, and it is divided into two categories: humans and robots. Humans include all the body parts that may be involved in manipulation, mainly the hands. Robots describe the hardware and software that affect the robot’s manipulation ability and characteristics, such as different types of mechanical arms, depth cameras, force sensors, end effectors.

#### 4.1.4. Task

The task layer is used to describe the task goals and processes in manipulation. Manipulation tasks are bound with specific entities, such as *Cut_Apple_a*, *Pour_Cola_a*, *Make_Coffee_a*, and *Insert_Key_a*. A manipulation task contains four properties: the initial state, final state, action sequence, and task object. The description of the state includes semantics, visual-scene graphs, and physical parameters, such as coordinates and postures. The action sequence is mainly based on a set of action primitives, as well as related objects, agents, and scenes. The task object refers to the objects included in the task, which are connected to corresponding entities in the object layer.

#### 4.1.5. Action

The action layer is used to describe the action primitives in manipulation. Action primitives are indivisible, and in human manipulation, they are the smallest semantic unit, while in robot manipulation, they are low-level short-term tasks that can be directly completed through motion planning. Serialized action primitives can be combined into tasks and skills. In order to define the action primitives in manipulation more accurately, they are annotated according to three factors: contact, force, and trajectory. The action primitives can be represented in five dimensions: contact type, contact duration, force direction, trajectory type, and motion periodicity. Based on the contact type, actions can be divided into rigid contact, soft contact, and non-contact. Based on the contact duration, actions can be classified into continuous contact and non-continuous contact. Based on the force direction, actions can be divided into inward force, outward force, and tangential force. Based on the trajectory type, actions can be classified into one-dimensional movement, two-dimensional movement, and three-dimensional movement. Depending on whether the motion trajectory has periodicity, actions can be divided into periodic motion and non-periodic motion. The properties of the action primitives are shown in Table 1. We list some of the action primitives considered in this work. 

#### 4.1.6. Skill

The skill layer is used to describe refined models of tasks that combine knowledge and experience. Skills are derived from tasks with similar actions, such as *Cut_Fruit*, *Pour_Water*, *Make_drink*, and *Peg-in-Hole*. In order to define the skills in manipulation more accurately, they are annotated in three dimensions: complexity, collaboration, and precision. Based on their complexity, skills can be classified into simple skills, consisting of up to three serialized action primitives, and complex skills, consisting of more than three serialized action primitives. Depending on whether tool collaboration is required, skills that can be completed independently by agents relying on their own abilities and functions are independent skills, while skills that require tool or end-effector coordination are collaborative skills. Based on precision, the skills involved in dexterous manipulation with high precision requirements are called fine-grained skills, while those with low precision requirements are called coarse-grained skills. In Table 2. We list some of the skills considered in this work, along with their properties.

**Figure 2:**
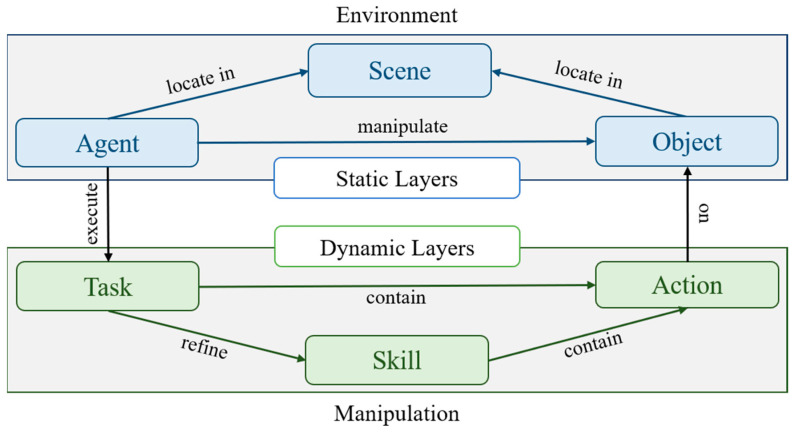
The hierarchical architecture of the knowledge-representation model.

We model knowledge representation according to six decoupled layers: scene, object, agent, task, action, and skill. Figure 2 shows the hierarchical structure. Specifically, the scene, object, and agent belong to the static environment, while the task, action, and skill belong to the dynamic manipulation.

### 4.2. Multi-Module Manipulative-Knowledge-Representation System

In terms of the defined questions, multi-layer knowledge can represent all factors in manipulation. In terms of system design, we adopted the latest graph database, neo4j [12], to build our hierarchical knowledge base. Compared with the Protégé [29] ontology schema based on RDF and OWL rules, neo4j’s property-graph schema weakens the logical constraints, which helps to store complex and varied attribute knowledge. Based on this schema, we built a multi-module knowledge-representation system, consisting of an ontology-knowledge module, a template-knowledge module, and an instance-data module.

#### 4.2.1. Ontology

The ontology module is a collection of abstract concepts, similar to a common-sense knowledge base. It is used to store semantic knowledge on the six layers, scene, object, agent, task, action, and skill, as well as the attribute labels of objects, actions, and skills. The ontology module only stores knowledge within the six layers and does not describe logical relations or temporal relations between layers other than the task layer. The ontology module is static and is only updated during knowledge inference and completion.

#### 4.2.2. Template

The template module describes manipulation processes that are centered around action sequences, with tasks or skills as units of knowledge. These include relations or temporal relations between different layers. Each manipulation task can be represented as a task template, and similar manipulation tasks can be represented as skill templates. Each node in each template is mapped from the corresponding node in the ontology. The template module is dynamic and can be flexibly added or removed.

#### 4.2.3. Instance

The instance module is the most active module, similar to a manipulation log. It can be understood as the database underlying the knowledge-representation system, adding specific execution parameters and timestamps based on the corresponding template. Each execution of each manipulation task is stored as an instance. Each node and relation in the instance are mapped from the corresponding node and relation in the template. The instance module is continuously updated during the repeated execution of manipulation tasks.

**Figure 3:**
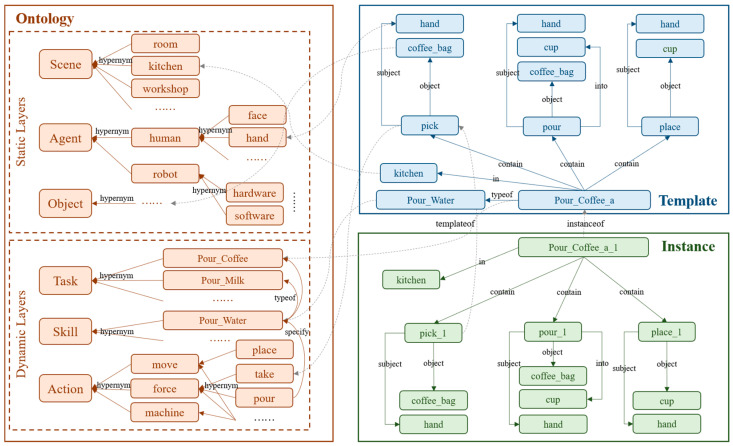
Modular architecture of the knowledge-representation system.

The knowledge-representation system was built based on three interrelated and specialized module structures: ontology, template, and instance. The overall structure is shown in Figure 3. The stability and flexibility of the knowledge-base architecture were ensured by this approach.

## 5. Semantic-Knowledge Extraction for Task-Centric Manipulation

The extraction of the semantic knowledge required for our knowledge base from different data sources was an important prerequisite task based on the knowledge-representation model and system we propose for robot manipulation. Existing knowledge bases for robots [2,3,5,6,7] were considered. Unfortunately, the task descriptions in these knowledge bases are flat and chaotic. Using a multi-layer and multi-module structure, knowledge was added to the template module as the core, an external knowledge base was connected to expand the knowledge in the ontology module and, finally, the knowledge in the instance module was supplemented during manipulation.

### 5.1. Data Collection

Step 1: Collection of manipulation task templates. The data were collected and annotated from wikiHow [30], an open-source life-guidance platform that is widely utilized. Guides were selected that describe real and uncomplicated manipulation tasks, which involve a physical displacement or state change in the subject or object during the process. One example is *how to cut an apple*. Each piece of wikiHow data comprises three parts: Title, Headline, and Text. The Title provides the task name, while the Headline offers the sequence of actions. Following the format specifications of RoboCSE [31], we extracted keywords from wikiHow that directly represented the task, action, agent, object, and scene, and added the respective suffixes, *.t*, *.a*, *.i*, *.o*, and *.l*. After this step, we obtained 317 manipulation-task templates, along with labeled data for actions, agents, objects, and scenes.

Step 2: Construction of knowledge triplets based on labeled data. Due to the discreteness of the labeled data in the manipulation task, action, and object, we considered combining them according to the rules to form a set of triplets that described the manipulation task, which was the data structure of the template module shown in Figure 3. After this step, we obtained 10,184 triplets, including 780 entities and 14 relations, corresponding to the template modules in the knowledge base.

Step 3: Linkage to DBpedia and retrieval of neighbors. The subject, object, and scene entities were linked to DBpedia through the SparQL (https://dbpedia.org/sparql/, (accessed on 28 February 2023)) query interface. The entities were used as head nodes, and the relation was restricted to *ingredient*, *region*, *main_ingredient*, and *hypernym* to retrieve the tail node in order to obtain the first-order neighbor information of the entities. After this step, 584 new triplets were excavated from the external knowledge base, mainly expanding the knowledge of the ontology module, including five new relations and one hundred and fifty-six new entities.

Step 4: Addition of unique identifiers to action entities. After completing the three steps above, we obtained sufficient knowledge that could be embedded. However, given that different actions with the same semantics may have different parameters in robot-manipulation tasks, if actions with the same name under different tasks point to the same entity, the data structure of the template module becomes confused. Therefore, we attached randomly generated hash suffixes to all the action entities in the template module to indicate their uniqueness. We also added action meta nodes to the ontology module, which were connected to specific actions in the template module through the *templateof* relation. Similarly, corresponding entities in the template and instance modules were connected through the *instanceof* relation. After this step, we achieved knowledge extraction in the ontology and template modules, while the data in the instance module were continuously added to the knowledge base during the robot manipulation.

### 5.2. Data Statistic

The statistics of the data scale of the knowledge base are presented in Table 3. In total, there were 10,768 triples, consisting of 936 entities and 21 relations. The full list of relations in the knowledge base is provided in Table 4. It should be noted that the relations listed here are only those of the external type and do not include the internal relations, such as he entity properties.

## 6. Knowledge-Graph Embedding with Graph Convolutional Networks

The knowledge-representation model and system for robot manipulation that were established, as well as the semantically extracted knowledge centered around the tasks, laid the foundation for the subsequent work. In this section, a knowledge-graph-embedding approach is proposed to transform the robot-manipulation factors into feature vectors, which facilitates subsequent robot-manipulation-task planning and object-oriented transfer.

Given a knowledge graph G, knowledge-graph embedding aims to represent entities E and relations R in a continuous feature space through vectors in different dimensions. Compared with traditional rule-based knowledge inference, vector operations can greatly simplify the problem of predicting relations that are missing from knowledge graphs. Let $F\left(e_{i}\right)$ represent the embedding vector of entity $e_{i}\in E$ and F(r) represent the embedding vector of relation r∈R. Classical knowledge-graph embedding methods, such as DistMult [32] and TransE [23], are based on embedding vectors to define the scoring function of the triple $\left(e_{i},\;r,\;e_{j}\right)$, so that greater numbers of correct triples obtain higher scores and ranks.

The typical multi-layer structure of our knowledge-representation model corresponds to six types of entity: scene, object, agent, task, action, and skill. We note that different types of entities are interrelated and influence each other. For example, the features of a task entity should include information related to its associated actions and objects, not just the task itself. Classical knowledge-graph-embedding methods do not consider information transmission between adjacent nodes, so we considered introducing graph convolutional networks (GCN). The application of GCNs is an effective graph-modeling method that naturally suits knowledge graphs. The core idea of graph convolutional networks is to fuse the features of neighboring nodes into the current node. The message-propagation framework of graph convolutional networks is:

(2) $$H^{l+1}=\sigma \left(D^{-\frac{1}{2}}AD^{-\frac{1}{2}}H^{l}W^{l}\right),$$

where D is the degree matrix, A is the adjacency matrix, W is a learnable network parameter, and σ is the activation function. We use the ReLU activation function. $H^{l}$ and $H^{l+1}$ are the nodes in the lth and l+1th layers of the graph convolutional network, respectively, which can also be understood as the hidden-layer embedding vectors of $e_{i}$ in the knowledge graph.

Classical GCNs are only applicable to isomorphic graph problems, while knowledge graphs contain a large number of different node and edge labels, which are heterogeneous graphs. For example, our knowledge representation included 936 entities and 21 relations. Inspired by RGCN [27], we divided the heterogeneous graph into several isomorphic graphs containing a single relation, and then applied the isomorphic-graph method to solve the problem. For each relation, both the inward and outward pointing nodes were considered as its neighboring points, and self-loop features were added, followed by feature fusion, to participate in the update of the central node:

(3) $$h_{i}^{l+1}=\sigma \left(\sum_{r\in R}\sum_{j\epsilon N_{i}^{r}}\frac{1}{c_{i,r}}W_{r}^{l}h_{j}^{l}+W_{0}^{l}h_{i}^{l}\right),$$

where R denotes the relation set and $N_{i}^{r}$ denotes the set of all the neighbors of node i with relation r. The $c_{i,r}$ is a problem-specific normalization constant. The σ is the ReLU activation function. The $h^{l}$ and $h^{l+1}$ are the nodes in the lth and l+1th layers of the heterogeneous graph convolutional network respectively, which can also be understood as the hidden embedding vectors of $e_{i}$ in the knowledge graph.

The number of layers in our graph convolutional network was set to two, which combined the first and second-order neighbor information of the central node. For the target entity $e_{i}$ in the knowledge graph, after aggregation in terms of the relations and neighboring nodes, we ultimately obtained the encoded embedding vector $F\left(e_{i}\right)$ that aggregated the full neighbor information.

Regarding the decoding phase, considering that our dataset has a small size and the relational structure has a clear directionality, our method differs from RGCN in that we use the scoring function of the knowledge-graph-embedding model, TransE, to calculate the scores of different triplets:

(4) $$f\left(s,r,o\right)=-\|h+r-t{\|}_{1/2}=-\|F\left(o^{\prime }\right)+r_{o^{\prime },o_{2}}-F\left(o_{2}\right){\|}_{1/2},$$

where $\|.{\|}_{1/2}$ denotes the $L_{1}$ and $L_{2}$ distances, F(.) denotes the feature vector of the entity fused with the neighboring nodes, and $r_{o^{\prime },o_{2}}$ denotes all the possible candidate relations between the two entities. Training is accomplished through cross-entropy loss:

(5) $$ℒ=\frac{1}{\left|S\right|}\underset{\left(h,r,\cdot \right)\in \varepsilon }{\sum }\left(\frac{1}{\left|\varepsilon \right|}\underset{t\epsilon \varepsilon }{\sum }y\left(h,r,t\right)\cdot logf\left(h,r,t\right)+\left(1-y\left(h,r,t\right)\right)\cdot log\left(1-f\left(h,r,t\right)\right)\right)$$

where S denotes the set of all the triplets in the knowledge graph and ℒ represents the average of all triple losses. For each triplet, the cross-entropy loss between the predicted value f(h,r,t) and the actual value y(h,r,t) of the tail node t is calculated. Here, y(h,r,t) was smoothed to a number between [0, 1] to reduce the risk of overfitting. The objective of this loss function is to minimize the difference between the predicted and actual values to improve the model’s performance.

**Figure 4:**
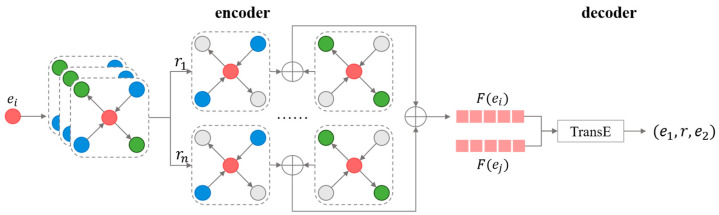
Overview of the knowledge-graph-embedding method based on graph convolutional networks.

A summary of the knowledge-graph-embedding method based on graph convolutional networks is shown in Figure 4. It consists of an encoder and a decoder. The encoder is a heterogeneous graph convolutional network (HGCN), and the decoder predicts the relation of the triplet through the knowledge-graph-embedding method, TransE. We describe the corresponding comparative experiments in Section 7.1 to demonstrate the superiority of our knowledge-graph-embedding method in our knowledge framework.

## 7. Experiments

Two different types of experiment were conducted in this work. Firstly, we verified the superiority of the proposed knowledge-graph-embedding method based on graph convolutional networks, which accurately extracts entity features and successfully predicts missing relations in our robot-manipulation knowledge-representation model. On the other hand, we inferred new manipulation-task templates based on the generated knowledge-graph embeddings, achieved object-oriented task transfer, and verified the feasibility of the method for real-world robot manipulation.

### 7.1. Knowledge-Graph Embedding in Robot Manipulation

#### 7.1.1. Dataset

The knowledge triplets were extracted from the knowledge-representation system. We ignored the multimodal data in the instance module and only retained the semantic knowledge stored in the ontology module and the template module, strictly following the triplet format of the knowledge graph. The entity properties were also added to the training data in the form of triplets. These included action properties, skill properties, and object properties, such as *(push*, *contactduration*, *continuous)* and *(coke_a*, *packing*, *can)*. The data consisted of 13,154 triplets, 3064 entities, and 36 relations. The dataset was split into a training set, a validation set, and a test set in a ratio of 12:1:1. The batch size was set to 2000 during the training, and the epoch was set to 10,000.

#### 7.1.2. Baselines

We evaluated two classic knowledge-graph-embedding models, DistMult [32] and TransE [23], as the baselines. These two baselines differ from our method in that they use fixed entity embeddings instead of the heterogeneous graph-embedding encoder. They focus on the learning structure and do not aggregate information from adjacent entities.

#### 7.1.3. Metrics

Mean Reciprocal Ranking (MRR) and Hit@N were used as metrics to measure the quality of the embeddings. Given a correct triplet, the head and tail entities of the triplet were replaced by every other entity, and the score of each triplet in the current embedding model was calculated after each replacement. The rankings of all the triplets resulting from the above replacements were recorded, and the rank of each correct triplet was noted. This process was repeated for all the triplets S in the test set, and the rank of each correct triplet $rank_{i}$ was recorded. Thus, the MRR and Hit@N were respectively defined as follows:

(6) $$MRR=\frac{1}{\left|S\right|}\sum_{i=1}^{\left|S\right|}\frac{1}{rank_{i}},$$

(7) $$Hit@N=\frac{1}{\left|S\right|}\sum_{i=1}^{\left|S\right|}I\left(rank_{i}\leq n\right),$$

where I is an indicator function.The higher the MRR and Hit@N results, the better the prediction.

#### 7.1.4. Results

The results for the filtered MRR and Hits@1, 3, and 10 in our manipulation-knowledge framework are presented in Table 5. It can be observed that our embedding method using heterogeneous graph convolutional networks as encoders and TransE as a decoder (HGCN + TransE) significantly outperforms other baseline methods. The Hits@1 (directly correct) prediction accuracy for the potential triplets is 65.7%, the accuracy for Hits@3 (correct among the top three) is 77.4%, and the accuracy of Hits@10 (correct among the top ten) is 87.9%. Compared to DistMult and TransE, our method adopts graph convolutional networks instead of word embeddings to generate embedded vectors. This allows the inclusion of more information, such as entity contextual information, relation information, and attribute information. This results in a more accurate expression of entities and relations, and an improvement in the accuracy of relation prediction, obtaining better results for the metrics MRR and Hit@1, 3, and 10.

### 7.2. Robot-Manipulation-Task Planning and Object-Oriented Transfer

Based on the knowledge-representation model and system, we used the above knowledge-graph-embedding methods to realize new-task planning based on known tasks, that is, object-oriented manipulation-task transfer.

#### 7.2.1. Definition

The experimental tasks are defined with reference to previous work on robotic manipulation task planning [33]. Assuming a fixed scene and a given manipulation task, if prior manipulative knowledge regarding the performance or demonstration of the task is available in the knowledge base, we retrieve the template and instance modules from the knowledge base, call the corresponding action sequences and parameters, and transfer them to the robot for execution. If there is no prior manipulative knowledge of the task in the knowledge base, but semantic knowledge of a similar task for other objects and the target object itself is available, our method can still be employed for task planning. Similar tasks refer to multiple tasks derived from the same skill template, with similar action sequences but different objects, such as *(Cut_Apple*, *Cut_Pear)* and *(Make_Coffee*, *Make_Milk)*.

**Figure 5:**
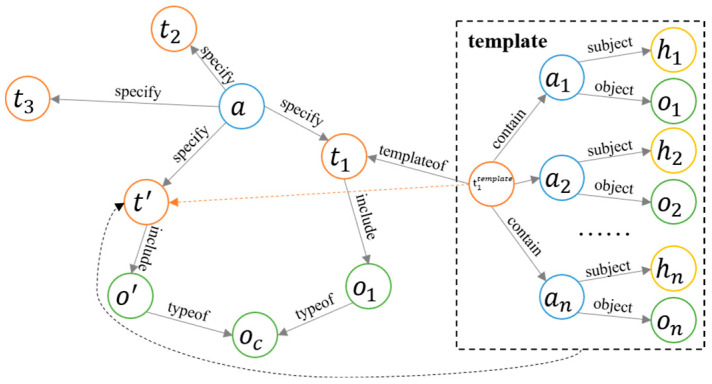
The process of object-oriented task transfer.

Considering that the variables in the planning are objects, the task planning is defined as an object-oriented task transfer, as shown in Figure 5. A task set $T=\left\{t_{1},t_{2},\ldots ,t_{n}\right\}$ is already present in the knowledge graph, and each task in the set is connected to its corresponding task template by a triplet $t_{i}^{template},templateof,t_{i}$. The $t^{\prime }$ is a new task entity, which is associated with the existing knowledge graph by the triplets $t^{\prime },include,o^{\prime }$ and $a,specify,t^{\prime }$, which represent prior information about the task $t^{\prime }$. Considering that the prior information associated with the new entity is often limited, it is necessary to infer more relevant linking information for the new entity. Our goal is to predict the action and manipulation sequences for $t^{\prime }$ based on the existing task templates. This goal can be transformed into a search for the task entity $t_{1}$ in the T that is the most similar to $t^{\prime }$ in terms of its features. Finally, the template $t_{1}^{template}$, which contains the motion parameters and action sequences, is copied and mapped to ${t^{\prime }}^{template}$, and all the object entities $o_{1}$ in $t_{1}^{template}$ are replaced by $o^{\prime }$ to obtain the final template for $t^{\prime }$.

#### 7.2.2. Task

We manipulated a real-world robot based on the predictions made in the knowledge framework. We set up three categories of tasks derived from simple skills *Pour_Water*, *Stir_Drink* and complex skills *Make_Drink*. Table 6 shows the task categories and success criteria.

**Figure 6:**
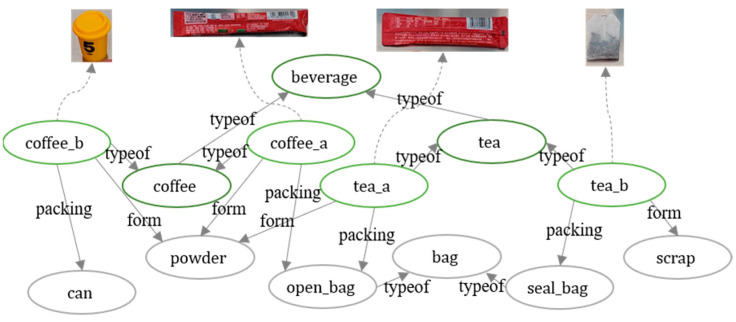
A knowledge graph of object properties in our experiments.

We used eighteen objects of five categories of drink, including coffee, tea, milk, juice, and soda. These objects were of different brands and had different forms and packing. Figure 6 gives examples of two different coffees and two different teas, respectively. They correspond to different object properties in the knowledge graph, such as form, which may be powder or scraps, and packing, which may be *seal_bag*, *open_bag*, or *can*. This prior knowledge changes the action sequence. It also affects the knowledge-graph embeddings and, thus, the existing templates chosen by the knowledge framework to make connections to new objects. A total of 33 task templates were derived from the task categories and objects. We selected three task templates from each task category. After the robot successfully manipulated the corresponding task instance, it was added to the knowledge base as prior knowledge. The remaining 24 task templates were used as test tasks. Each task template underwent five trials, for a total of one hundred and twenty task instances.

#### 7.2.3. Environment

**Figure 7:**
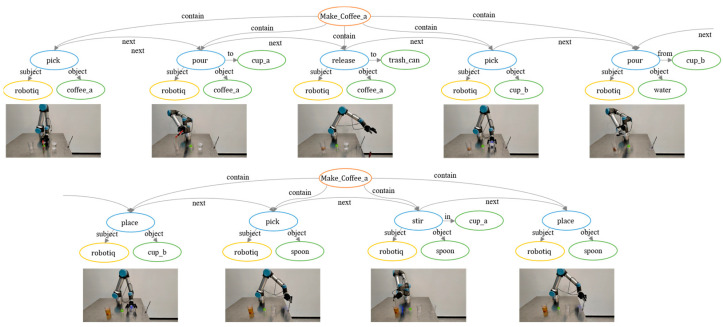
A complete task on our real-world manipulation platform.

A fixed manipulation platform, a UR5 robot arm, and a Robotiq gripper were used in this work. There were four fixed initial positions on the platform: *make*, *drink1*, *drink*2, and *spoon*. Figure 6 shows the manipulation environment and a complete task template derived from *Make_Drink*. Each primitive action contained prior knowledge of subject, object, and object complement, and bound the physical parameters of the target state. The low-level motion planning of the action process was handled by the RRT planner. A visual-recognition module was not added, as the focus of this experiment was on predicting the action sequence and manipulation sequence, and deviations in object detection would have lowered the accuracy of the manipulation tasks. Therefore, in this experiment, it was assumed that object labels and positions were given. Figure 7 gives an example of a manipulation task in this experimental environment.

#### 7.2.4. Results

Three types of baseline were selected in this experiment. The first was an end-to-end Seq2seq [34] model, which used the task templates in the knowledge base as the training data to directly generate action sequences based on the task names. The second was rule-based matching, which selected the optimal matching template by calculating the shortest path between the task nodes to generate semantic action sequences and manipulation sequences. The embedding-based method selected the optimal matching template by generating feature vectors to produce semantic action sequences and manipulation sequences.

Table 7 reports the accuracy of the action-sequence prediction and robot execution. The Seq2Seq did not query the knowledge base and relied solely on text training and prediction, so it cannot predict manipulation sequences that contain motion parameters. Furthermore, due to the inadequate sizes of the templates, the accuracy of the action-sequence prediction was also low. We believe that with the support of large amounts of data, the accuracy would increase substantially. The rule matching utilized the graph structure of the knowledge base to predict and execute manipulation sequences based on the existing templates, and had a significantly higher accuracy than Seq2Seq. The embedding-based method integrated more implicit features in addition to the knowledge-base utilization, thus demonstrating the best performance. In particular, the incorporation of HGCN in our proposed method improved the accuracy of the embedding representation and, hence, the accuracy of action sequence. Based on the correct action sequence, the execution accuracy was also correspondingly improved. The experiment showed that our proposed knowledge-based planning method can operate real robots more effectively than other methods. However, due to errors in the grasping position and object pose in the real world, the accuracy of robot execution is significantly lower than the accuracy of the action-sequence prediction using our method.

## 8. Conclusions

In this paper, a semantic robot-manipulation framework with a knowledge graph was introduced. The core of the framework is a multi-layer knowledge-representation model consisting of manipulation scenes, objects, agents, tasks, actions, and skills, as well as a knowledge-representation system consisting of ontology, templates, and instances. The extraction of manipulation knowledge from different data sources was achieved based on task templates. Next, a learning-based knowledge-graph-embedding method was proposed to provide accurate feature information for manipulation-task planning and transfer.

To evaluate the proposed framework, we designed a knowledge-graph-embedding comparative experiment to evaluate the prediction accuracy of our method. Finally, the feasibility and significance of the knowledge framework for object-oriented task transfer were verified through robot-manipulation experiments in a real-world environment. In future work, expanding the knowledge base with more data sources in different modalities is planned, considering multi-modal fusion in knowledge-graph embedding, and focusing on the clustering and extraction of skills from manipulation tasks.

## Figures and Tables

**Table 1:** Examples of action properties.

Action Properties		Move	Surround	Push	Pull	Lift	Insert	Press	Rotate
Contact Type	Rigid			○	●	●	●	○	●
	Soft			○				○	
	Non	●	●						
Contact Duration	Continuous			●	●	●	●	○	●
	Non-continuous							○	
Force Direction	Inward			●			●	●	
	Outward				●	●			
	Tangential								●
Trajectory type	One			●	●	●	●	●	
	Two		●						●
	Three	●							
Periodicity	Periodic		●						●
	Aperiodic	●		●	●	●	●	●	

○ indicates that this action primitive has multiple possible classifications in the current dimension. ● indicates that this action primitive has only a unique classification in the current dimension.

**Table 2:** Examples of skill properties.

Skill Properties		Cut_Fruit	Pour_Water	Make_Drink	Peg-in-Hole
Complexity	Simple		●		●
	Complex	●		●	
Collaboration	Cooperative	●		●	●
	Native		●		
Precision	Fine-grained				●
	Coarse-grained	●	●	●	

● indicates the classification of this skill in the current dimension.

**Table 3:** Data statistics from the knowledge base.

Source	Triple	Relation	Entity				
			Task	Action	Agent	Object	Scene
wikiHow DBpedia	10,768	21	317	57	8	548	6

**Table 4:** External relations in the knowledge base.

Module	Relation	Definition	Example
Template Instance	Subject	The agent of an action.	take.a: human.i
	Object	The object on which the action acts.	place.a: knife.o
	From	The object of the action is derived from the target object.	take.a: refrigerator.o
	Into	The action object enters the target object.	pour.a: cup.o
	Next	The sequence of actions.	take.a: place.a
	Contain	The actions that constitute a task.	Saute_Vegetable.t: take.a
	Start	The action at the start of the task.	Wipe_The_Cupboard.t: open.a
	End	The action at the end of the task.	Empty_The_Bench.t: place.a
	On	The action object is above the target object.	place.a: bookshelf.o
	With	The action depends on the target object.	wash.a: brush.o
	In	The action object takes place inside the target object/scene.	place.a: bathroom.l
	Beside	The action takes place next to the target object.	place.a: sink.o
	Under	The action takes place below the target object.	wash.a: water_tap.o
	To	The action takes the target object as the destination.	move.a: telephone.o
Ontology	Typeof	Categories of entities.	chair.o: furniture.o
	Hypernym	Hierarchy of entities.	hand.i: human.i
	Include	Tasks contain objects.	Saute_Vegetable.t: vegetable.o
	Specify	An action specifies a task.	wipe.a: Wipe_The_Cupboard.t
	Ingredient	The components of an object.	kettle_corn.o: salt.o
Cross-module	Templateof	Connect similar entities in ontology and template.	place_KwEcyO.a: place.a
	Instanceof	Connect similar entities in template and instance.	place_KwEcyO.a_1: place_KwEcyO.a

**Table 5:** The results for filtered MRR and Hits@1, 3, and 10.

Model	MRR	Hits@		
		1	3	10
DistMult	0.341	0.209	0.412	0.592
TransE	0.405	0.250	0.495	0.705
HGCN + TransE(ours)	0.731	0.657	0.774	0.879

**Table 6:** The task categories and success criteria.

**Task Category**	**Success Criteria**
Pour_Water	whether a robot tilts to pour a drink into a cup
Stir_Drink	whether a robot inserts a spoon into a drink and moves it periodically
Make_Drink	whether a robot makes a drink using a mixture of two drinks

**Table 7:** The results of the test of the accuracy of action-sequence prediction and execution.

Method		Action Sequence Prediction	Robot Execution
Seq2seq		0.292	/
Rule Matching		0.625	0.533
Embedding	DistMult	0.792	0.642
	TransE	0.833	0.717
	HGCN + TransE(ours)	0.917	0.808

## Data Availability

Our related datasets are publicly available at https://github.com/TsingM/Semantic-Knowledge-Extraction (accessed on 16 March 2023).
